# Glycosylation Contributes to Thermostability and Proteolytic Resistance of rFIP-nha (*Nectria haematococca*)

**DOI:** 10.3390/molecules28176386

**Published:** 2023-08-31

**Authors:** Yusi Liu, Tamara Hoppenbrouwers, Yulu Wang, Yingying Xie, Xue Wei, Haowen Zhang, Guoming Du, Khandader Md Sharif Uddin Imam, Harry Wichers, Zhen Li, Shanna Bastiaan-Net

**Affiliations:** 1 Laboratory of Biomanufacturing and Food Engineering, Institute of Food Science and Technology, Chinese Academy of Agriculture Sciences, Beijing 100193, China; wnewyx@163.com (Y.W.); 15210650462@163.com (Y.X.); weixue15701202366@163.com (X.W.); haowen237@hotmail.com (H.Z.); dgmcaas@163.com (G.D.); biotech_shakil@hotmail.com (K.M.S.U.I.); lizhen19890911@163.com (Z.L.); 2 Wageningen Food and Biobased Research, Wageningen University and Research, 6708 WG Wageningen, The Netherlands; tamara.hoppenbrouwers@wur.nl (T.H.); harry.wichers@wur.nl (H.W.); 3 Laboratory of Food Chemistry, Wageningen University, 6708 WG Wageningen, The Netherlands; 4 Laboratory of Food Quality and Design, Wageningen University, 6708 WG Wageningen, The Netherlands; 5 Beijing SeekGene BioSciences Co., Ltd., Beijing 102206, China

**Keywords:** immunomodulatory proteins, glycosylation, structure, thermostability, digestion resistance

## Abstract

Glycosylation is an important post-translational modification of proteins, contributing to protein function, stability and subcellular localization. Fungal immunomodulatory proteins (FIPs) are a group of small proteins with notable immunomodulatory activity, some of which are glycoproteins. In this study, the impact of glycosylation on the bioactivity and biochemical characteristics of FIP-nha (from *Nectria haematococca*) is described. Three rFIP-nha glycan mutants (N5A, N39A, N5+39A) were constructed and expressed in *Pichia pastoris* to study the functionality of the specific N-glycosylation on amino acid N5 and N39. Their protein characteristics, structure, stability and activity were tested. WT and mutants all formed tetramers, with no obvious difference in crystal structures. Their melting temperatures were 82.2 °C (WT), 81.4 °C (N5A), 80.7 °C (N39A) and 80.1 °C (N5+39A), indicating that glycosylation improves thermostability of rFIP-nha. Digestion assays showed that glycosylation on either site improved pepsin resistance, while 39N-glycosylation was important for trypsin resistance. Based on the 3D structure and analysis of enzyme cleavage sites, we conclude that glycosylation might interfere with hydrolysis via increasing steric hindrance. WT and mutants exerted similar bioactivity on tumor cell metabolism and red blood cells hemagglutination. Taken together, these findings indicate that glycosylation of FIP-nha impacts its thermostability and digestion resistance.

## 1. Introduction

Glycosylation is one of the most important post-translational modifications (PTMs), playing a decisive role in controlling protein function and diversity [1]. In general, more than 85% of proteins are glycosylated [2]. Based on the amino acid residues to which the glycan is linked to, protein glycosylation could be classified into N-glycosylation, O-glycosylation, C-glycosylation, S-glycosylation and P-glycosylation [3]. N-glycosylation is a conserved process of protein modification that eukaryotic cells use to guide protein folding, assembly and trafficking [4]. The N-linked glycans (also called “oligosaccharide trees”) are attached onto asparagine (or less commonly, arginine) residues within the primary protein sequence of N/R-X-S/T/C, where X is any amino acid except proline [3]. The coupled oligosaccharides can be distinguished into three main types based on the coupled saccharide residues: high mannose types, complex type oligosaccharides and hybrid versions [5]. The high mannose-type glycosylation is common seen in heterologous expression, where a gene is recombinantly expressed in a host organism in which it does not occur naturally [5].

Many studies have indicated that glycosylation is important for protein stability and biofunctionality, such as half-life, thermostability, hydrolysis resistance, catalytic activity and cellular cytotoxicity [1]. For instance, programmed death ligand-1 (PD-L1) is primarily N-glycosylated and interacts extracellularly with programmed cell death protein-1 (PD-1), leading to tumor-associated immune escape [6]. The half-life of glycosylated PD-L1 is at least four-times longer than that of non-glycosylated ones, indicating that glycosylation enhances PD-L1’s protein stability [6]. Han et al. improved the enzyme activity and thermostability of the β-1,4-endoglucanase from *Chaetomium thermophilum* by introducing an additional N-glycosylation site and optimizing of N-glycosylation sites to reduce entropy [7]. Regarding hydrolysis resistance, N-glycosylation improved the pepsin resistance of histidine acid phosphatase phytases and the trypsin resistance of β-glucuronidase by increasing steric hindrance around the peptide backbone [8]. All monoclonal antibodies (MAbs) typically have a single N-glycosylation site on each of the Fc region, which correlates with their clinical efficacy and half-life [9]. The N-glycans of MAbs are involved in binding with Fc receptors and inducing antibody-dependent cellular cytotoxicity [9,10]. Meanwhile, the removal of N-glycans might lead to a shorter half-life of MAbs in the blood circulation [10]. 

Fungi have long been a source of food (in the form of edible mushrooms) and medicines [11]. Fungal immunomodulatory proteins (FIPs) are a group of low-molecular-weight (MW) proteins investigated as potential anti-tumor drugs [12,13]. To date, eight native or recombinant forms of FIPs exist as glycoproteins, and over 20 FIPs have potential glycosylation sites [14]. However, little research has been done toward the impacts of glycosylation on the bioactivity of FIPs. FIP-nha (from *Nectria haematococca*) contains two glycosylation sites at positions N5 and N39. In 2013, Bastiaan-Net et al. expressed rFIP-nha in *P. pastoris*, which resulted in three bands on SDS-PAGE, representing the non-glycosylated, single-site glycosylated and fully glycosylated rFIP-nha respectively [15]. It remains unclear if glycosylation affects rFIP-nha’s biochemical characteristics and bioactivity. In this study, we constructed glycosylation mutants of FIP-nha and analyzed their stability and bioactivity. To gain insights into the stability-determining mechanism of rFIP-nha, crystal structures of the wild-type (WT) and mutants were solved and analyzed, which will provide a better understanding of the mechanisms involved in the PTM-structure–activity relationship and of the potential of glycosylation to regulate stability.

## 2. Results and Discussion

### 2.1. Biochemical Characteristics of Glycosylation Mutants of FIP-nha

**Figure 1:**
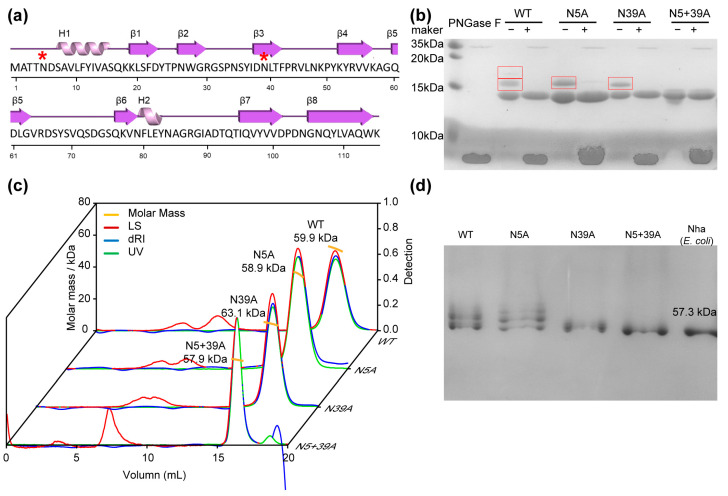
Glycosylation mutants of rFIP-nha and their characteristics. (**a**) Secondary structure of rFIP-nha shown in cartoon form, with H indicating α-helix, β indicating β-sheets, connected lines indicating loops and * indicating N-glycosylation sites; (**b**) WT and mutants (around 2 μmol protein loaded) shown on 15% SDS-PAGE with or without PNGase F treatment, with the glycosylated bands highlighted with a red box; (**c**) SLS analysis of WT and mutants; (**d**) WT and mutants shown on 10% native-PAGE gel (1 μmol protein loaded) with *E. coli*-expressed FIP-nha.

In our previous study, we showed that FIP-nha could be glycosylated at either or both N5 and N39 sites, and all the variants were heterologously expressed in *P. pastoris* strain X-33 [15]. However, the level of glycosylation by *P. pastoris* is not absolute. The rFIP-nha are produced as a mixture of unglycosylated, single- and double-glycosylated forms. Attempts to separate these three glycosylation forms via size-exclusion and ion exchange chromatography were unsuccessful and pointed to the formation of multimers, as previously observed for rFIP-nha produced in *E. coli* [16]. To study the impact of glycosylation on functionality of rFIP-nha, two single mutants (N5A and N39A) and one double mutant (N5+39A) were constructed to remove glycosylation sites. The double-glycosylated wild-type is denominated by WT. The SDS-PAGE results showed that the purified WT, N5A and N39A were partially glycosylated, while the N5+39A variant was indeed unglycosylated (Figure 1b). The glycans could be removed by PNGase F treatment under denaturing conditions (Figure 1b), which confirmed the N-glycosylation pattern. Apart from FIP-nha, several other FIPs are glycoproteins or have potential N-glycosylation sites, some of which were glycosylated in heterologous expression systems. For instance, the immunomodulatory proteins from *Poria cocos* (PCP) and *Antrodia camphorata* (ACA) are glycoproteins [17,18]; also in addition, transfected FIP-gts from *Ganodera tsugae* was expressed as a glycoprotein in S21 cells [19]. 

SLS and native-PAGE results suggested that rFIP-nha and its variants were all homogeneously tetrameric in the solution (Figure 1c,d). This was identical to what was observed for rFIP-nha expressed by *E. coli,* which was used in the gel as an MW reference (Figure 1d) [16]. The tetrameric form, in combination with the unsuccessful separation of the different glycosylation forms under native conditions via size exclusion or ion-exchange chromatography, seems to indicate that the tetramers consisted of both glycosylated and non-glycosylated FIP-nha forms. Separation under denaturing/reducing conditions might cause structural or activity changes afterward, so we preferred mutants to investigate the glycosylation impacts of rFIP-nha. 

**Figure 2:**
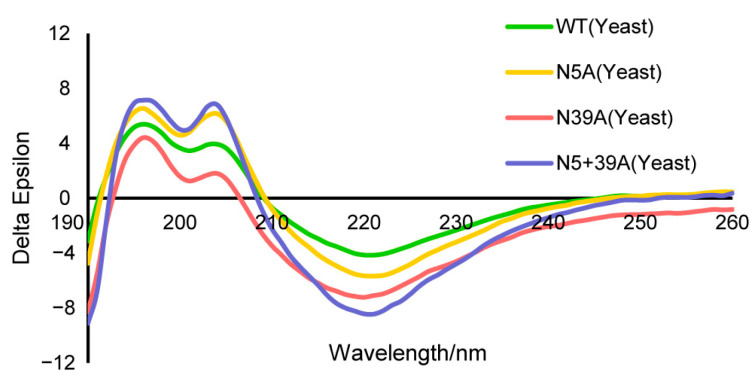
CD analysis of rFIP-nha and its variant.

The secondary protein structures of WT and mutants were analyzed with circular dichroism (CD). The results were similar, indicating that the amino acid mutation and glycosylation had little impact on the secondary structure of rFIP-nha (Figure 2, Table 1).

### 2.2. Crystal Structure Analysis

**Figure 3:**
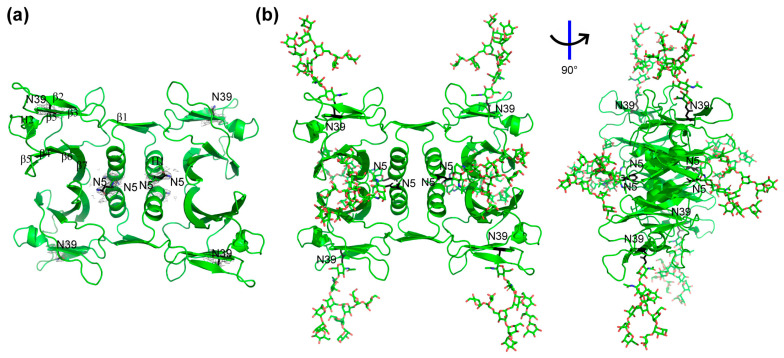
Tetrameric structure of rFIP-nha and its simulated glycosylated form with N5 and N39 labeled in black. (**a**) Crystal structure of rFIP-nha (WT) with secondary structure labeled in one chain and with glycosylation sites N5 and N39 showed electron density in grey; (**b**) structure of rFIP-nha with N-glycosylation (O shown in red and N shown in blue), which was predicted and simulated via GlyProt (http://www.glycosciences.de/modeling/glyprot/php/main.php; accessed on 20 December 2021).

To determine the impact of glycosylation on tertiary and quaternary structural features of rFIP-nha, we attempted to obtain the crystal structures of all FIP-nha variants. However, we only managed to obtain crystals and solved the structures of WT, N39A and N5+39A (Figure S1, Table S1). Since all structures turned out to be almost identical to each other, we used the structure of the WT as an overall model for the analysis. Unfortunately, none of the structures contained a density indicative of N-glycosylation. The lack of glycosylation in our crystal structures may have been due to the nonuniformity of glycosylated protein or the flexibility of glycan chains [20]. Specifically, the composition, as analyzed on SDS-PAGE, visualized that the double-glycosylated band of WT represented only 6.7%, while the single glycosylated and un-glycosylated bands accounted for 31.7% and 61.6%, respectively (Figure 1b). Removal of the un-glycosylated form by size exclusion chromatography was only possible using denaturation/reduction of the tetramers, which would possibly bias the crystallizations afterwards. As no carbohydrate densities were observed in the protein crystals generated, they were simulated by GlyProt to allow further analysis [21]. For the simulation, the GlcNAc_2_Man_9_ glycan structure was used, as this high mannose-type glycosylation modification is commonly expressed in *P. pastoris* [22]. Figure 3 shows the obtained crystal structure of WT (Figure 3a) and its predicted glycosylated form (Figure 3b). Based on its crystal structure, N5 is at the start of the N-terminus just in front of the characteristic α-helix and located in the middle of the tetrameric structure. N39 is positioned in the middle of the third β-sheet and located at the four corners of the tetrameric structure.

### 2.3. Thermostability of rFIP-nha and Its Variants

**Figure 4:**
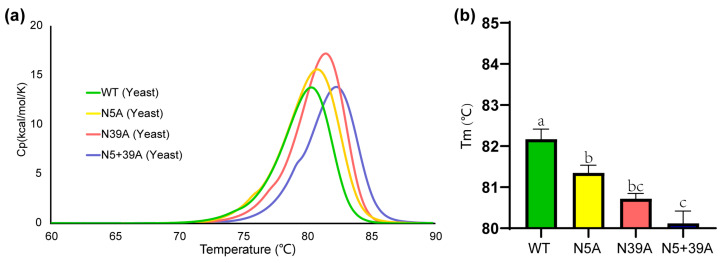
Thermostability of rFIP-nha and its variants. (**a**) Specific heat capacity at a constant pressure (Cp) of rFIP-nha tested via DSC with a temperature increase; (**b**) statistical analysis histogram of the T_m_ value with a, b, c shown the significant difference.

To check the impact of glycosylation of rFIP-nha on thermostability, a differential scanning calorimetry (DSC) study was performed. The T_m_ values for WT, N5A, N39A and N5+39A were 82.2 °C, 81.4 °C, 80.7 °C and 80.1 °C, respectively (Figure 4). Highly glycosylated forms of rFIP-nha seemed more stable, indicating that N-glycosylation might contribute to thermostability. Several other studies have also suggested that N-glycosylation is of relevance for protein thermostability. For instance, introducing N-glycosylation at Q258N/Q349N of phytase increased its T_m_ [23]. As mentioned in the introduction, by optimizing its N-glycosylation sites, the thermostability of a hyper-thermostable endoglucanase was increased [7]. In addition, the removal of N-glycosylation from exo-inulinase (*Kluyveromyces cicerisporus*) led to a decrease in T_m_ [24]. Glycosylation may directly affect the protein folding process by reducing the conformational freedom of the local peptide backbone and thus reduce the loss of configurational entropy on folding [25]. Also, the carbonyl groups of the N-glycan saccharides could change polarity and donate hydrogen bonds which can enhance protein thermostability [26].

### 2.4. Proteolytic Resistance

**Figure 5:**
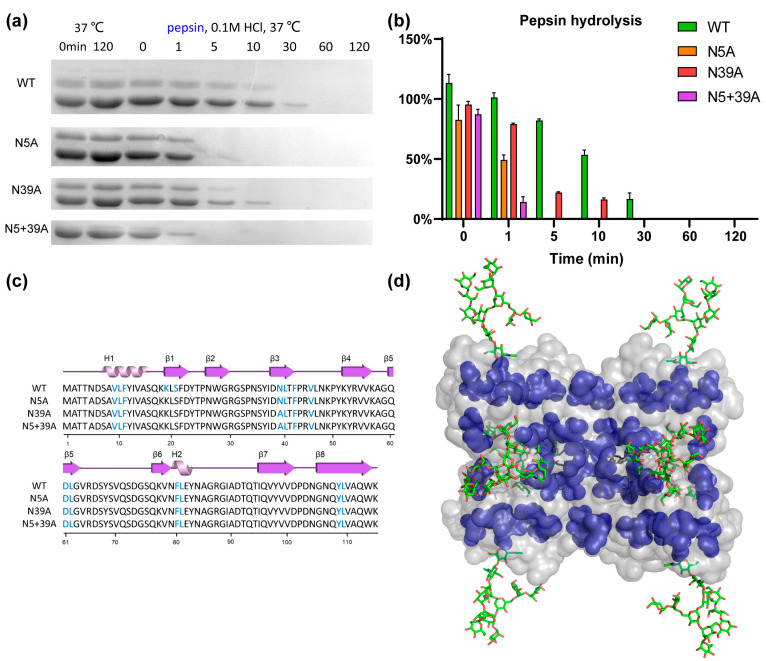
Pepsin resistance of rFIP-nha. (**a**) rFIP-nha and its variants exposed to 0.4 mg/mL (100 U/mL) pepsin digestion at pH 1.3, 37 °C; (**b**) quantification of protein amount during pepsin digestion with non-treated samples at 0 min as control; (**c**) pepsin cleavage sites shown on the amino acid sequence of rFIP-nha and its variants, colored in blue; (**d**) pepsin cleavage sites shown on rFIP-nha (WT) depicted in blue.

**Figure 6:**
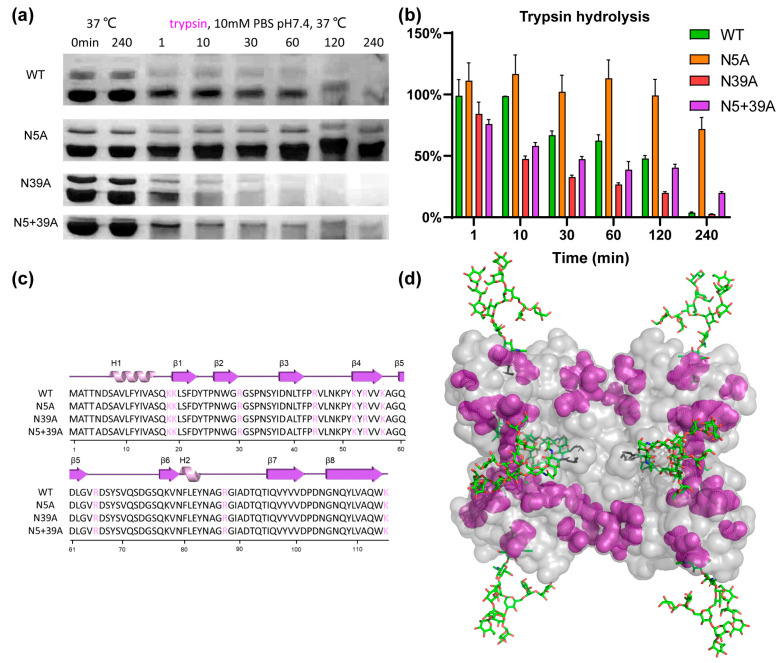
Trypsin resistance of rFIP-nha. (**a**) rFIP-nha and its variants exposed to 30 mg/mL (15,000 U/mL) trypsin digestion at 10 mM PBS pH 7.4, 37 °C; (**b**) Quantification of protein amount during trypsin digestion with non-treated samples at 0 min as control; (**c**) trypsin cleavage sites shown, colored in purple, on the amino acid sequence of rFIP-nha and its variants; (**d**) trypsin cleavage sites, depicted in purple, shown on rFIP-nha (WT).

To analyze the impact of glycosylation on proteolytic stability, WT and mutants were exposed to pepsin and trypsin. As shown in Figure 5, the stability of rFIP-nha during pepsin digestion was in this order: WT (T_1/2_ 11.6 min) > N39A (T_1/2_ 1.9 min) > N5A (T_1/2_ 1.3 min) > N5+39A (T_1/2_ 0.5 min). Double glycosylation significantly enhanced the pepsin resistance. Resistance to trypsin digestion is shown in Figure 6: N5A (T_1/2_ longer than 240 min) > WT (T_1/2_ 89.3 min) > N5+39A (T_1/2_ 13.9 min) > N39A (T_1/2_ 8.1 min). Interestingly, 39N-glycosylation contributed to trypsin resistance, while 5N-glycosylation did not contribute to this resistance. The results of pancreatin digestion correlated with trypsin digestion (Figure S2), which can likely to attributed to trypsin being the main protease in pancreatin [27]. As shown in Figure 1, rFIP-nha and its mutants existed as tetramer in solution. The tetramers of WT, N5A and N39A are mixtures which consist of glycosylated and unglycosylated forms, which means that the N-glycosylation could have an impact on almost all tetramers and affect their hydrolysis resistance. Several studies have indicated that the N-glycosylation of proteins contributes to proteolytic resistance. The IgY antibody became more flexible and disordered, as well as more sensitive to pepsin after de-glycosylation [28]. With N-glycosylation modification, β-mannanase’s thermal stability, pH stability, pepsin resistance and trypsin resistance were significantly improved compared to the non-glycosylated form [29]. Also, compared with un-glycosylated matriptase, the β 1-6 GlcNAc matriptase was more resistant to trypsin digestion, while, at the same time, an N-glycosidase-F treatment greatly reduced its resistance to degradation [30]. 

To better understand the mechanism of glycosylation impact on the observed differences in hydrolysis resistance among the rFIP-nha variants, pepsin and trypsin cleavage sites were highlighted on rFIP-nha. As shown in Figure 5c and Figure 6c, there were no differences in pepsin or trypsin cleavage sites of rFIP-nha and its variants. As shown in the 3D structure (Figure 5d), pepsin cleavage sites were uniformly distributed over the whole structure (around both glycosylation sites). Thus, both glycan chains appeared to be able to shield pepsin cleavage sites, possibly by steric hindrance, explaining the observed decrease in pepsin resistance in all mutants. Figure 6d shows that trypsin cleavage sites less surrounded the 5N-glycosylation position, and N5A positively enhanced the trypsin resistance of rFIP-nha; hence, the 5N-glycan might affect trypsin activity in a more special way. Perhaps trypsin activity near position 39N is diminished by allosteric regulation when position 5N is not occupied by a glycan chain. However, this is speculative and needs to be further investigated in the future. There are several studies that support our hypothesis that glycosylation improves hydrolysis resistance. A study of β-mannanase suggested the carbohydrate chain to shield protease sites, thereby enhancing its pepsin resistance [29]. Replacing the pepsin cleavage sites in the immediate vicinity of the N-glycosylation could improve pepsin resistance of phytases when produced in *E. coli* but had no effect on the pepsin resistance of N-glycosylated enzymes produced in *P. pastoris* [8], indicating that glycosylation can reduce pepsin’s accessibility to its cleavage sites. In another study, the presence of glycans on glucuronidase could increase steric hindrance around the peptide backbone of the enzyme and safeguard the enzyme from proteolytic hydrolysis [31].

### 2.5. Bioactivity of FIP-nha and Its Variants

To test the impact of glycosylation on the bioactivity of FIPs, human lung adenocarcinoma A549 and H2347 cells and human gastric cancer SGC7901 cells were exposed to rFIP-nha and its variants. As previously described, FIP-nha could be a promising candidate to develop as an anti-tumor drug [32,33,34]. However, there were no significant differences in tumor mitochondrial metabolism inhibition among the rFIP-nha variants toward the tested tumor cell lines (Figure S3). From this, we conclude that glycosylation (by *P. pastoris*) has no obvious impact on the direct tumor inhibition of rFIP-nha. Regarding hemagglutination activity, WT and mutants showed a slight difference on hemagglutination of mouse red blood cells, and no obvious differences were observed on the hemagglutination of red blood cells of guinea pig (Table S2 and Figure S4). WT induced the aggregation of mouse red blood cells when its concentration exceeded 0.125 µM, which is comparable to previous results [15]. However, inhibiting glycosylation at either position only increased the hemagglutination potency of rFIP-nha mutants slightly.

There is very little research discussing the effects of glycosylation on FIPs’ bioactivity, while some studies have shown that glycosylation has no impact on the biological activities of lectins [35,36,37]. Frutalin (a plant lectin) showed cytotoxicity on a broad panel of human cancer cells, and its cytotoxicity was glycosylation-independent [36]. For *Curcuma longa* rhizome lectin (CLA), the recombinant lectin expressed in *E. coli* and *P. pastoris* (glycosylated) behaved similar to native CLA in the agglutination of rabbit erythrocytes [37]. It is worth mentioning that Li et al. constructed LZ-8 with N-glycosylation, and the glycosylated form showed higher anti-inflammatory activity, while the secondary structure changed obviously between WT and the mutants with N-glycosylation [38]. Therefore, it could not be confirmed whether the observed bioactivity changes were caused by N-glycosylation or the structural changes of the mutants.

## 3. Materials and Methods

### 3.1. Materials and Chemicals

The plasmid pPICZαA and *P. pastoris* X-33 strain used for protein expression were purchased from Invitrogen (Carlsbad, NM, USA) and Biolab (Beijing, China), respectively. Electroporation device and 0.2 cm cuvettes were purchased from Bio-Rad (Carlsbad, NM, USA). The Ni-NTA agarose and Superdex 200 chromatography column for protein purification were purchased from QIAGEN (Dusseldorf, Germany) and GE healthcare, respectively. PNGase F was purchased from New England Biolabs (Ipswich, MA, USA). A549 cells, H2347 cells and SGC7901 cells were obtained from the Cell Bank of National Biomedical Laboratory, Chinese Academy of Medical Sciences (Beijing, China). Pepsin and trypsin were purchased from Solarbio (Beijing, China). Other chemicals were purchased from Biolab (Beijing, China).

### 3.2. Gene and Mutagenesis Construction of FIP-nha

The gene encoding FIP-nha (GeneBank: XM3043608) and mutations (for which the asparagine on either position 5 (N5A), position 39 (N39A) or both (N5+39A) was replaced by an alanine and synthesized by BaseClear B.V. (Leiden, the Netherlands). Originally, the genes were designed to contain a flexible linker and a myc-tag as previously described [15], but for this study, both were removed by PCR using the following primers: F 5′-CCGGAATTCGCTACCACCAATGAC; R 5′-TGCTCTAGATCAATGATGATGATGATGATGCTTCCACTG. The resulting PCR fragment was cut using the restriction enzymes EcoRI and XbaI (underlined) and inserted in pPICZαA using T4 DNA ligase (TaKaRa, Kusatsu, Japan). Plasmid pPICZαA carrying the gene of FIP-nha or its mutants was transformed into *P. pastoris* X-33. The rFIP-nha and its mutants contain a C-terminal His-tag for purification purposes. X33 transfection was performed following the protocol of pPICZαA via the electroporation and selection of transformants. 

### 3.3. Protein Preparation

Transformed X-33 colonies were cultured and fermented following the method adapted from the fermentation protocol of pPICZαA. Briefly, selected colonies were fermented in 100 mL BMGY media, shaking in 2 L bottles using an IS-RDS4 incubator shaker (SH Scientific, Compton, CA, USA) at 30 °C for 16 h to an OD600 of 4. The cells were collected by centrifugation (1500× *g*, 5 min, RT), resuspended in 400 mL BMMY in the same bottle and then fermented at 28 °C for 3 days, adding 1.5% (*v*/*v*) methanol every day. The rFIP proteins secreted into the growth medium were purified using Ni-TNA agarose (QIAGEN, Hilden, Germany) followed by Superdex 200 (1.6 cm × 20 cm, GE healthcare) size exclusion chromatography. The composition and purity of rFIP-nha were analyzed by a 15% reduction in SDS-PAGE (Tris-Glycine gel) and Image J, with around 2 μmol protein loaded and protein marker P1017–1 (LABLEAD, Beijing, China). Protein concentrations (in 10 mM PBS solution; pH 7.4) were quantified by the BCA kit (LABLEAD, Beijing, China), and all samples were snap-frozen by liquid nitrogen and stored at −80 °C.

### 3.4. Oligomerization State Analysis

The oligomerization state of rFIP-nha and its variants in solution was determined via static light scattering (SLS; DynaPro, WYATT technology, Asker, Norway). Then, 100 μL rFIP-nha or its variants (concentration >1 mg/mL in 10 mM PBS pH 7.4) were injected onto a Superdex 200 chromatography column, with LS signal, UV signal and dRI signal detection. Moreover, 10 mM PBS (pH 7.4) was used as an elution buffer with a flow rate of 0.45 mL/min. The molecular weight of each protein was calculated after the ASTRA software analysis.

### 3.5. Thermostability Measurements

The T_m_ of rFIP-nha and its variants was determined via a MicroCal PEAQ-DSC instrument. Concentrations of 1.0–2.0 mg/mL of proteins in 10 mM PBS (pH 7.4) were used for the DSC study. The T_m_ values of rFIP-nha and its variants were measured at a rate of 3 °C/min from 20 °C to 90 °C [39].

### 3.6. Protein Crystallization, X-ray Data Collection, Structure Determination and Refinement

Rough screening for protein crystallization was performed in 24-well plates at 20 °C under 864 reservoir conditions, including the PACT, JSCG (QIAGEN), Hampton Research kit and Wizard classic crystallization series (Rigaku, Cedar Park, TX, USA), using a hanging-drop diffusion method. Each drop contained 1 μL of reservoir buffer and 1 μL of protein (rFIP-nha WT 4.8 mg/mL; N5A 8.5 mg/mL; N39A 8.3 mg/mL; N5+39A 5.6 mg/mL). The optimized condition for WT crystallization consisted of 0.2 M (NH_4_)_2_SO_4_, 0.1 M NaAc pH 4.3 and 20% *w*/*v* PEG 1,000; the optimized condition for N39A crystallization consisted of 0.1 M NaCl, 0.1 M Bis-Tris pH 6.5, 1.5 M (NH_4_)_2_SO_4_; while the optimized condition for N5+39A crystallization consisted of 0.1 M NaCl, 0.1 M Bis-Tris pH 6.0, 1.5 M (NH_4_)_2_SO_4_. Despite the many reservoir conditions tested, we were unable to obtain an N5A crystallization with a high-resolution ration to determine its structure. The best crystals were preserved in liquid nitrogen and transferred to Shanghai Synchrotron Radiation Facility beamline BL19U and BL02U (Shanghai, China) for high-resolution diffraction data collection. The collected data was processed using the HKL2000 package [40]. Next, the crystal structures of WT and mutants were deciphered using the structure of rFIP-nha expressed by *E. coli* (PDB code: 7WDL) through the molecular replacement method using CCP4 software (version 7.0.066) [41]. Standard refinement was achieved using PHENIX (version 1.15.2) [42] and WinCoot (version 0.8.9) [43]. The glycosylation sites within the WT crystal structure were predicted via GlyProt (http://www.glycosciences.de/modeling/glyprot/php/main.php; accessed on 20 December 2021). Interfaces of rFIP-nha were calculated using CCP4 [41]. The hydrogen (H) bonds and salt bridges were predicted by PISA (https://www.ebi.ac.uk/msd-srv/prot_int/pistart.html; accessed on 20 November 2021). Structural graphics images were prepared with PyMOL (http://www.pymol.org; accessed on 15 October 2021).

The structural factors and coordinates for the rFIP-nha structures were deposited in the Protein Data Bank (rFIP-nha WT PDB code: 8GO5; rFIP-nha N39A PDB code: 8GO6; rFIP-nha N5+39A PDB code: 8GO7).

### 3.7. Digestion Resistance Measurements

Pepsin digestion of rFIP-nha and its variants was determined in 0.1 M HCl with 100 U/mL pepsin at 37 °C, with around 1 mg/mL of proteins in 10 mM PBS (pH 7.4), and the pH was measured via Metrohm 913 (Boston, MA, USA). The digestion was stopped by adjusting the pH beyond 7 by adding 0.5 M NaOH. The samples were collected at time points between 0 and 120 min. Trypsin digestion of rFIP-nha and its variants was determined in 10 mM PBS (pH 7.4) with 15,000 U/mL trypsin at 37 °C, with around 2 mg/mL of proteins. The digestion was stopped by incubation at 100 °C for 10 min. Samples were collected between 0 and 240 min. Pancreatin digestion of rFIP and its variants was determined in 10 mM PBS (pH 7.4) with 250 U/mL pancreatin at 37 °C, with around 1.5 mg/mL of proteins. The digestion was stopped by incubation at 100 °C for 10 min. Again, samples were collected between 0 and 240 min. The maintaining protein was analyzed by a 15% reduction of SDS-PAGE (Tris-Glycine gel) and Image J. The half resistant time (T_1/2_) of rFIP-nha was calculated via Excel with suitable trendline (R^2^ > 0.95). The pepsin and trypsin cleavage sites were predicted via Peptide_cutter (https://web.expasy.org/peptide_cutter/; accessed on 15 December 2021). The unit activities reported for pepsin, trypsin and pancreatin were according to the manufacturer’s stated lot specifications. 

### 3.8. Bioactivity Measurements

To determine direct antitumor cytotoxicity, A549 cells, H2347 cells or SGC7901 cells were seeded in a 96-well flat-bottom cell culture-treated plate at a density of 1 × 10^4^ per well and cultured in RPMI 1640 (Biological Industries, Beijing, China) supplemented with 10% fetal bovine serum (Biological Industries, Beijing, China), penicillin (100 U/mL) and streptomycin (100 μg/mL) at 37 °C in a humidified atmosphere with 5% CO_2_. After 12 h of culture, the cells were exposed to WT and mutants with a concentration of 0–4 μM for 24 h. Cell viability was determined using the CCK-8 cell proliferation and cytotoxicity assay kit (Biolab, Beijing, China) following the manufacturer’s instructions. Each experiment was repeated three times, and the viability of the non-treated control group was set to 100%.

The whole blood in the Alsever of mouse and guinea pig was ordered from Envigo (Venray, The Netherlands). The method of hemagglutination assay was clearly described before [35], with the small modification that the rFIP-nha concentration was calculated via molarity and started from 1 μM to 0.007813 μM. Concavalin A from *Canavalia ensiformis* (Sigma-Aldrich, Darmstadt, Germany) started from 20 μg/mL to 0.1563 μg/mL, and 10 mM PBS (pH 7.4) were used, respectively, as positive and negative controls. 

### 3.9. Statistics

Comparison between T_m_ of rFIP-nha and its variants were calculated with one-way ANOVA with Multiple Comparisons using GraphPad Prism. Comparison between the cell viability tests of tumor cell lines were calculated with two-way ANOVA with Multiple Comparisons using GraphPad Prism. A value of *p* < 0.05 was considered to be significant.

## 4. Conclusions

In this study, we characterized several aspects of the impact of glycosylation on the anti-tumor protein FIP-nha. rFIP-nha and its glycosylation defective mutants (N5A, N39A and N5+39A) were constructed and recombinantly expressed in *P. pastoris* strain X-33. There was a correlation between the level of glycosylation and thermostability. In addition, glycosylation enhanced its proteolytic resistance, which may have been caused by steric hindrance, as N-linked glycans seemed to prevent enzymes’ accessibility of pepsin- and trypsin-cleavage sites. Glycosylation had no apparent impact on the direct tumor inhibition and hemagglutination potency of rFIP-nha. Our results suggest that glycosylation contributes to thermostability and digestion resistance. Regarding the future use of FIP-nha as an antitumor drug, glycosylated protein would be recommended due to its higher stability, and injection would be preferable to oral treatment to avoid gastrointestinal degradation.

## Figures and Tables

**Table 1:** Secondary structure of rFIP-nha and its variant via CD analysis.

FIP-nha	Helix	Antiparallel	Parallel	Beta-Turn	Random Coil	Total Sum
WT	6.3%	48.5%	3.7%	16.5%	29.3%	104.3%
N5A	6.4%	48.1%	3.7%	16.5%	29.4%	104.1%
N39A	6.4%	46.9%	3.7%	16.7%	29.8%	103.6%
N5+39A	6.4%	46.8%	3.7%	16.6%	30.0%	103.5%

## Data Availability

We clarify all data described in the manuscript are located.

## Supplementary Materials

The following supporting information can be downloaded at: https://www.mdpi.com/article/10.3390/molecules28176386/s1, Table S1: Crystal parameters, data collection, and structure refinement of rFIP-nha (WT, N39A, N5+39A); Figure S1: Structure alignment of WT (green), N39A (magenta) and N5+39A (cyan); the N-glycosylation sites shown in sticks form; Figure S2: Pancreatin digestion of rFIP-nha and its variants; Figure S3: Direct antitumor effects of rFIP-nha; Table S2: Hemagglutination of rFIP-nha variants on red blood cells of mouse and guinea pig; Figure S4: Hemagglutination of red blood cells upon exposure to rFIP-nha variants.
