# Supplementary material for: Glycosylation Contributes to Thermostability and Proteolytic Resistance of rFIP-nha (Nectria haematococca)
Source: Molecules. 2023 Aug 31;28(17):6386. doi: 10.3390/molecules28176386 (PMC10490071; doi:10.3390/molecules28176386)
Supplement: Supplementary file 1 [file molecules-28-06386-s001.zip › molecules-2569495-supplementary.pdf]

Supplementary Materials

Table S1. Crystal parameters, data collection, and structure refinement of rFIP-nha (WT, N39A, N5+39A).

	WT	N39A	N5+39A
**PDB code**	8GO5	8GO6	8GO7
**Data collection**			
Space group	*P 1*	*C 1 2 1*	*C 1 2 1*
Cell dimensions			
a, *b*, *c* (Å)	66.577, 74.467, 87.473	135.251, 75.284, 89.615	118.311, 101.304, 74.877
	99°, 112°, 109°	90°, 132°, 90°	90°, 129°, 90°
Resolution (Å)	25.63 - 2.809 (2.909 - 2.809)*	33.56 - 2.813 (2.914 - 2.813)*	28.29 - 2.303 (2.385 - 2.303)*
No. of measured reflections	33042	15611	29132
No. of unique reflections	2950	1502	2925
R_{sym} or R_{merge}	0.082 (0.320)	0.153 (0.773)	0.117 (0.470)
$I / \sigma I$	13.91 (2.41)	15.06 (2.34)	29.89 (3.34)
Completeness (%)	96.82 (85.76)	94.29 (92.60)	95.96 (97.83)
Redundancy	2.4 (2.5)	6.3 (6.8)	6.3 (6.3)
**Refinement statistics**			
Resolution (Å)	50.00 - 2.81	50.00 - 2.81	50.00 - 2.30
No. reflections	32985	15575	29103
R_{work} / R_{free} (%)	21.87/25.51	19.63/24.97	21.57/25.74
No. atoms			
Protein	6995	3403	3370
Ligand/ion	0	0	0
Water	0	0	105
B-factors (Å^2)			
Protein	67.04	46.07	54.34
Ligand/ion	-	-	-
Water	-	-	54.92
R.m.s. deviations			
Bond lengths (Å)	0.009	0.008	0.007
Bond angles (°)	1.05	0.9	0.87
Ramachandran plot statistics			
Most favored (%)	98.85	96.21	97.84
Allowed (%)	1.15	3.55	2.16
Disallowed (%)	0.40	7.3	0

*Values in parentheses are for highest-resolution shell.

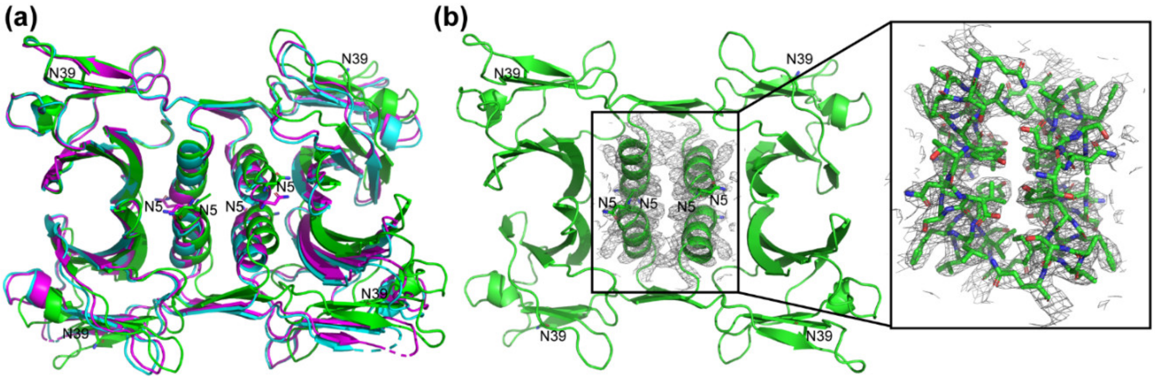

Figure S1. Structure of WT, N39A and N5+39A. (a) Structure alignment of WT (green), N39A (magenta) and N5+39A (cyan) with the N-glycosylation sites shown in sticks form; (b) Structure of WT with N-terminal shown with electron density in cartoon and sticks form.

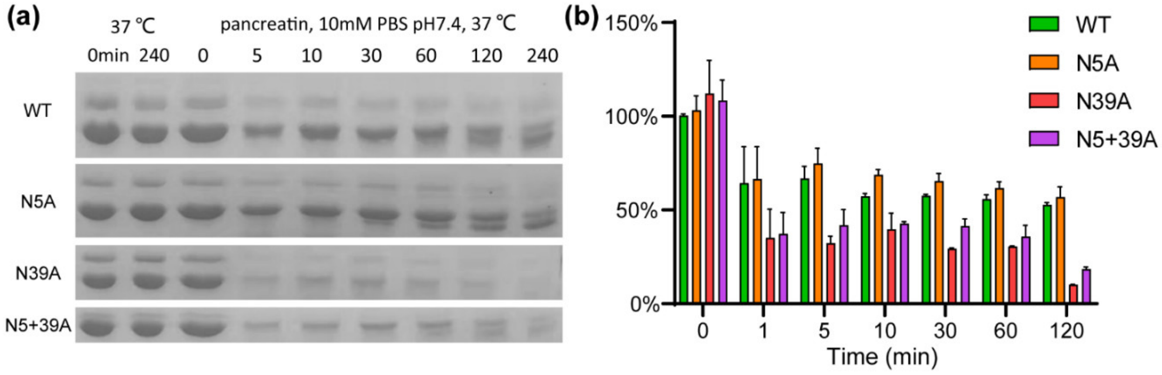

Figure S2. Pancreatin digestion of rFIP-nha and its variants. (a) 1 mg/mL pancreatin digestion on rFIP-nha and its variants at 10mM PBS pH 7.4, 37 °C; (b) Quantification of protein amount within pancreatin hydrolysis with non-treated samples at 0 min as control

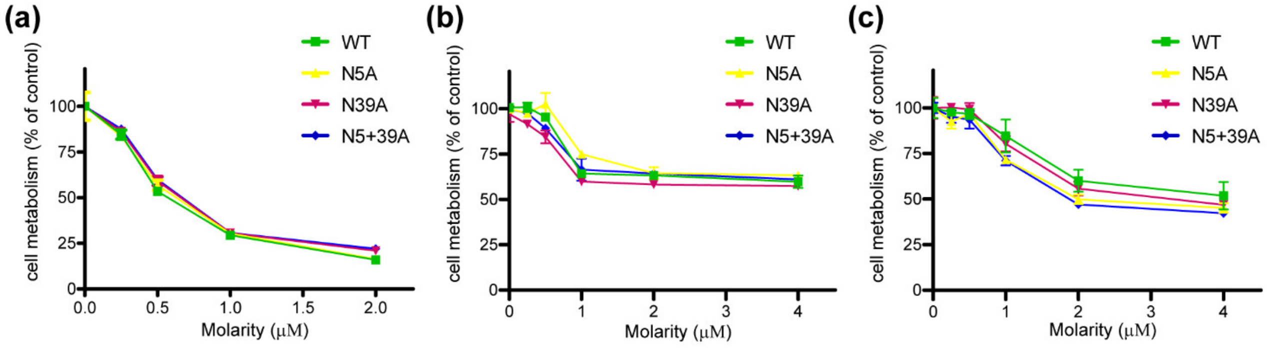

Figure S3. Direct antitumor effects of rFIP-nha. Cell metabolism of rFIP-nha and its variants on (a) A549 cells, (b) H1975 cells, (c) SGC 7901 cells.

Table S2. Hemagglutination of rFIP-nha variants on red blood cells of mouse and guinea pig.

	Red blood cells of mouse	Red blood cells of guinea pig
WT	≥0.125 μM	≥0.125 μM
N5A	≥0.0625 μM	≥0.125 μM
N39A	≥0.0625 μM	≥0.125 μM
N5+39A	≥0.0625 μM	≥0.125 μM
PBS	-	-
ConA-IV	≥5 μg/mL	≥5 μg/mL

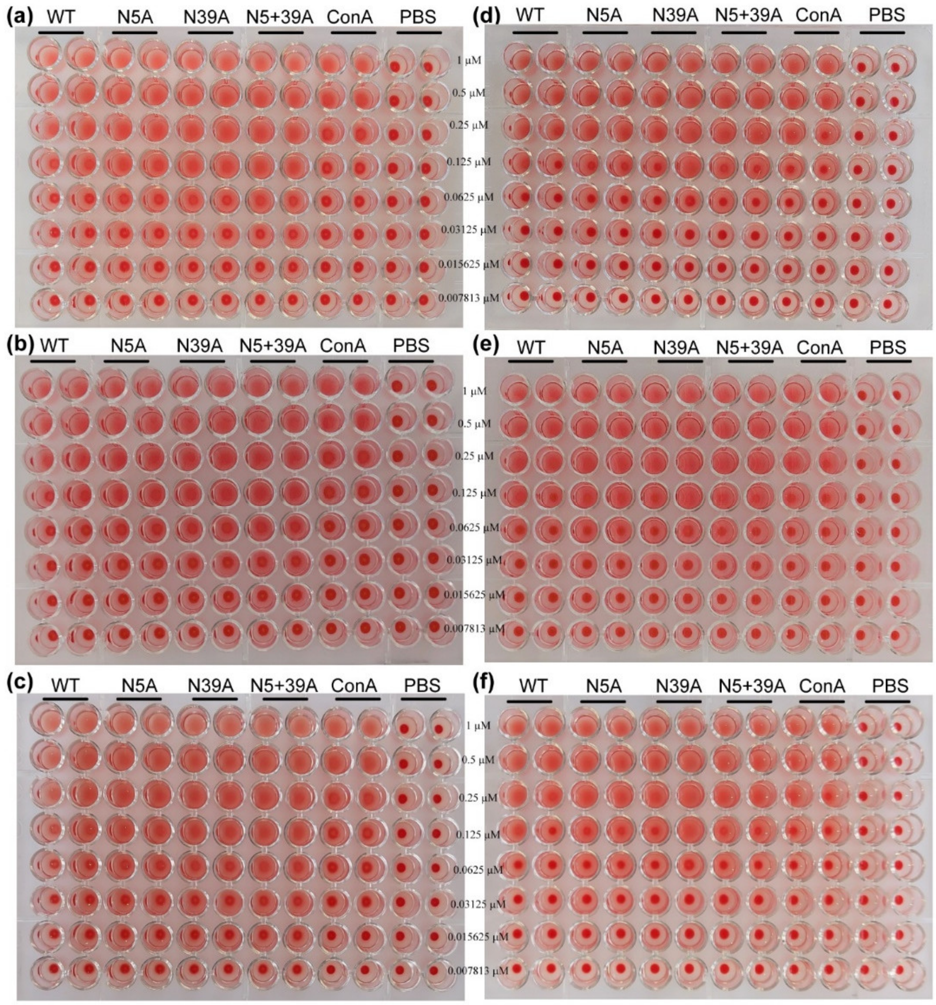

Figure S4. Hemagglutination of red blood cells upon exposure to rFIP-nha variants. (a), (b), (c) show hemagglutination of mouse erythrocytes after 2h, 4h, 16h, respectively; (d), (e), (f) show hemagglutination of guinea pig erythrocytes after 2h, 4h, 16h, respectively. rFIP-nha concentration was calculated via molarity and started from 1 μM to 0.007813 μM. Concavalin A from *Canavalia ensiformis* started from 20 μg/mL to 0.1563 μg/mL and10 mM PBS (pH7.4) were used respectively as positive and negative controls.
